# Generation of neutral and high-density electron–positron pair plasmas in the laboratory

**DOI:** 10.1038/ncomms7747

**Published:** 2015-04-23

**Authors:** G. Sarri, K. Poder, J. M. Cole, W. Schumaker, A. Di Piazza, B. Reville, T. Dzelzainis, D. Doria, L. A. Gizzi, G. Grittani, S. Kar, C. H. Keitel, K. Krushelnick, S. Kuschel, S. P. D. Mangles, Z. Najmudin, N. Shukla, L. O. Silva, D. Symes, A. G. R. Thomas, M. Vargas, J. Vieira, M. Zepf

**Affiliations:** 1 School of Mathematics and Physics, The Queen's University of Belfast, Belfast BT7 1NN, UK; 2 The John Adams Institute for Accelerator Science, Blackett Laboratory, Imperial College London, London SW7 2BZ, UK; 3 Center for Ultrafast Optical Science, University of Michigan, Ann Arbor, Michigan 48109-2099, USA; 4 Max-Planck-Institut für Kernphysik, Saupfercheckweg 1, 69117 Heidelberg, Germany; 5 Istituto Nazionale di Ottica, Consiglio Nazionale delle Ricerche, 56124 Pisa, Italy; 6 INFN, Sez. Pisa, Largo B. Pontecorvo, 3-56127 Pisa, Italy; 7 Helmholtz Institute Jena, Fröbelstieg 3, 07743 Jena, Germany; 8 GoLP/Instituto de Plasmas e Fusão Nuclear, Instituto Superior Técnico, Universidade de Lisboa, Lisbon, Portugal; 9 Central Laser Facility, Rutherford Appleton Laboratory, Didcot, Oxfordshire OX11 0QX, UK

## Abstract

Electron–positron pair plasmas represent a unique state of matter, whereby there exists an intrinsic and complete symmetry between negatively charged (matter) and positively charged (antimatter) particles. These plasmas play a fundamental role in the dynamics of ultra-massive astrophysical objects and are believed to be associated with the emission of ultra-bright gamma-ray bursts. Despite extensive theoretical modelling, our knowledge of this state of matter is still speculative, owing to the extreme difficulty in recreating neutral matter–antimatter plasmas in the laboratory. Here we show that, by using a compact laser-driven setup, ion-free electron–positron plasmas with unique characteristics can be produced. Their charge neutrality (same amount of matter and antimatter), high-density and small divergence finally open up the possibility of studying electron–positron plasmas in controlled laboratory experiments.

## Abstract (web summary)

Electron–positron pair plasma—a state of matter with a complete symmetry between negatively and positively charged particles—are found in many astrophysical object. Here, the authors use high-power laser to create an ion-free electron–positron plasma in the laboratory.

## Graphical abstract

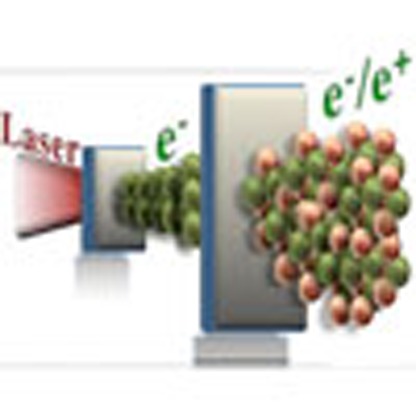

Electron–positron (e^−^/e^+^) plasmas are emitted, in the form of ultra-relativistic winds or collimated jets, by some of the most energetic or powerful objects in the Universe, such as black holes^1^,^2^, pulsars^3^ and quasars^4^. These plasmas are associated with violent emission of gamma-rays in the form of short-lived (milliseconds up to a few minutes) bursts, which are among the most luminous events ever observed in the Universe. These phenomena represent an unmatched astrophysical laboratory to test physics at its limit and, given their immense distance from Earth (some more distant than several billion light years), they also provide a unique window on the very early stages of our Universe^5^,^6^,^7^. Arguably, one of the most intriguing questions is how these gamma-ray bursts are produced. It is generally accepted that gamma-ray bursts should arise from synchrotron emission of relativistic shocks generated within an electron–positron beam^8^,^9^. This radiative mechanism requires a strong and long-lived (
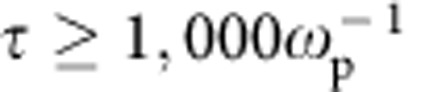
, with *ω*_p_ being the electron–positron plasma frequency) magnetic field; however, Weibel-mediated shocks generate magnetic fields that should decay on a fast timescale 
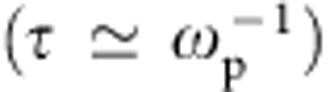
 due to phase-space mixing^9^. Also, diffusive Fermi acceleration, a proposed candidate for the acceleration of cosmic rays^9^, requires magnetic field strengths that are much higher than the average intergalactic magnetic field (≃nT)^10^. These and other questions could be addressed by *ad hoc* laboratory experiments; however, the extreme difficulty in generating e^−^/e^+^ populations that are dense enough to permit collective behaviour^11^,^12^ is still preventing laboratory studies and the properties of this peculiar state of matter are only inferred from the indirect interpretation of its radiative signatures and from matching numerical models. The intrinsic symmetry between negatively charged (e^−^) and positively charged (e^+^) particles within the plasma makes their dynamics significantly different from that of an electron-ion plasma or from a purely electronic beam. In the first case, the mass symmetry of the oppositely charged species induces different growth rates for a series of kinetic and fluid instabilities^13^, and significantly affects the possibility of generating acoustic or drift waves. In the second case, the overall beam neutrality forbids the generation of current-driven magnetic fields that would hamper the onset of transverse instabilities.

Different schemes have been proposed for the laboratory generation of e^−^/e^+^ plasmas: in large-scale conventional accelerators, the possibility of recombining high-quality electron and positron beams via magnetic chicanes^14^ is envisaged and a different approach is foreseen in confining low-energy positrons using radioactive sources with Penning traps^11^,^15^. The proposed APEX experiment^12^ builds on this idea, accumulating a large number of positrons in a multicell Penning trap, before injection into a stellarator plasma confinement device. The major challenge of these schemes is the recombination of these separate electron and positron populations. Alternative schemes have been proposed in which electrons and positrons are generated *in situ*^16^,^17^,^18^,^19^,^20^,^21^, thus avoiding the aforementioned recombination issues. Despite the intrinsic interest of these results, the low percentage of positrons in the electron–positron beam (of the order, if not <10%) and the low-density reported (collision-less skin depth much greater than the beam size, forbidding plasma-like behaviour) prevent their application to the laboratory study of e^−^/e^+^ plasmas. All these previous experimental attempts have thus not been able to generate e^−^/e^+^ beams that present charge neutrality and a plasma-like behaviour, both fundamental pre-requisites for the laboratory study of this state of matter^14^.

We report here on the first experimental evidence of the generation of a high-density and neutral electron–positron plasma in the laboratory. Its high density 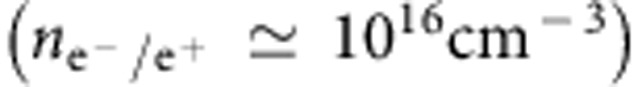 implies that the collision-less skin depth in the plasma is smaller than the plasma transverse size effectively allowing for collective effects to occur. These characteristics, together with the charge neutrality, small divergence 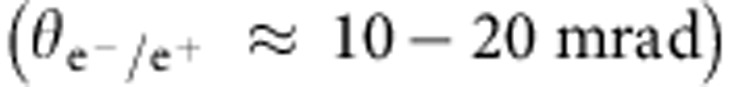, and high average Lorentz factor (*γ*_AV_≈15 with a power-law spectral distribution, comparable to what observed in astrophysical jets^22^) finally open up the possibility of studying the dynamics of e^−^/e^+^ plasmas in a controlled laboratory environment.

## Results

### Experimental setup

**Figure 1:**
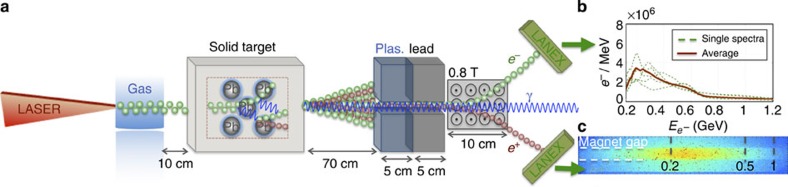
Experimental setup. (**a**) The laser wakefield-accelerated electrons (green spheres) impact onto a solid target, initiating a quantum electrodynamic cascade involving electrons, positrons (red spheres) and photons (blue sinusoids). The escaping electrons and positrons are separated and spectrally resolved using a magnetic spectrometer (details in the text) and a pair of LANEX screens. Plastic and lead shielding was inserted to reduce the noise on the LANEX screens as induced by both the low-energy electrons and gamma-rays generated, at wide angles, during the laser–gas and electron–solid target interactions. (**b**) Typical measured spectra of the electron beam without the solid target. Dashed green lines depict single-shot electron spectra, whereas the solid brown line is an average over five consecutive shots. (**c**) Typical positron signal, as recorded by the LANEX screen, for 0.5 cm of Pb. The image is to scale. The white dashed lines depict the projection of the magnet gap, whereas the grey dashed lines depict the position of 0.2, 0.5 and 1 GeV positrons on the LANEX screen.

The experiment (shown schematically in Fig. 1a) was carried out using the ASTRA-GEMINI laser system at the Rutherford Appleton Laboratory^23^, which delivered a laser beam with a central wavelength *λ*_L_=0.8 μm, energy on target *E*_L_≈14 J and a duration of *τ*_L_=42±4 fs. An *f*/20 off-axis parabola focussed this laser beam (focal spot with full-width half-maximum (27±3 μm) containing ∼60% of the laser energy, resulting in a peak intensity of ≃3 × 10^19^ W cm^−2^) onto the edge of a 20-mm-wide supersonic He gas jet doped with 3.5% of N_2_. A backing pressure of 45 bar was found to be optimum in terms of maximum electron energy and charge of the accelerated electron beam as resulting from ionization injection^24^,^25^ in the gas jet. Optical interferometry of the laser–gas jet interaction indicates this gas–pressure to correspond to a plasma density of *n*_pl_=(6.0±0.2) × 10^18^ cm^−3^. This interaction produced a reproducible electron beam (shot-to-shot fluctuation in charge and maximum energy below 10%) with a broad spectrum with maximum energy of the order of 600 MeV, full-width half-maximum divergence of 2 mrad and an overall charge of (0.3±0.1) nC, corresponding to (1.9±0.6) × 10^9^ electrons (see Fig. 1b for typical electron spectra and their average). This electron beam was then directed onto a Pb solid target of different thicknesses covering multiples of the material's radiation length (*d*=0.5, 1, 1.5, 2, 2.5, 3 and 4 cm, given that the radiation length for Pb is *L*_rad_≈0.5 cm (ref. 21)). The electrons and positrons escaping from the rear side of the target were then separated and spectrally resolved by a magnetic spectrometer. The details of this detector can be found in the Methods section.

### Experimental results

**Figure 2:**
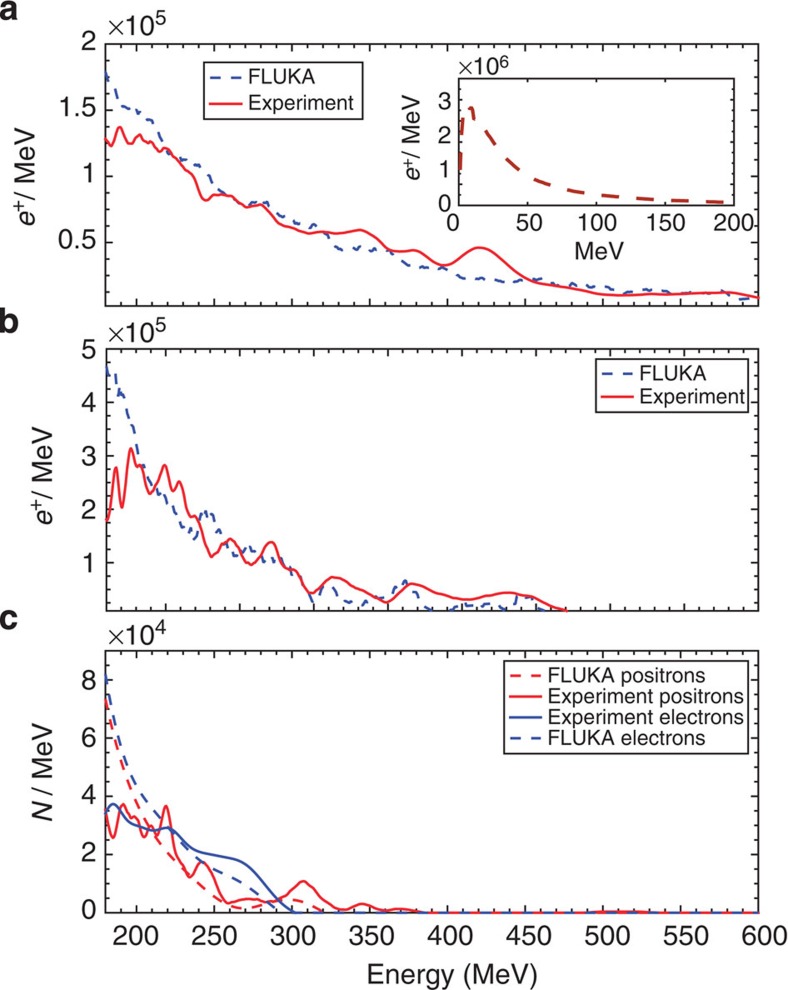
Positron spectra. Measured positron spectra, as resulting from the average over five consecutive shots, (solid lines) compared with that obtained from FLUKA simulations (dashed lines) for *d*=5 mm (**a**), *d*=2 cm (**b**) and *d*=4 cm (**c**). In this latter case, also the spectrum of the electrons escaping the target is plotted. Its similarity with the positron spectrum is a clear indication of the generation of a neutral electron–positron pair beam. The inset in **a** shows the simulated positron spectrum at low energy for *d*=5 mm, indicating a Jüttner–Synge distribution.

**Figure 3:**
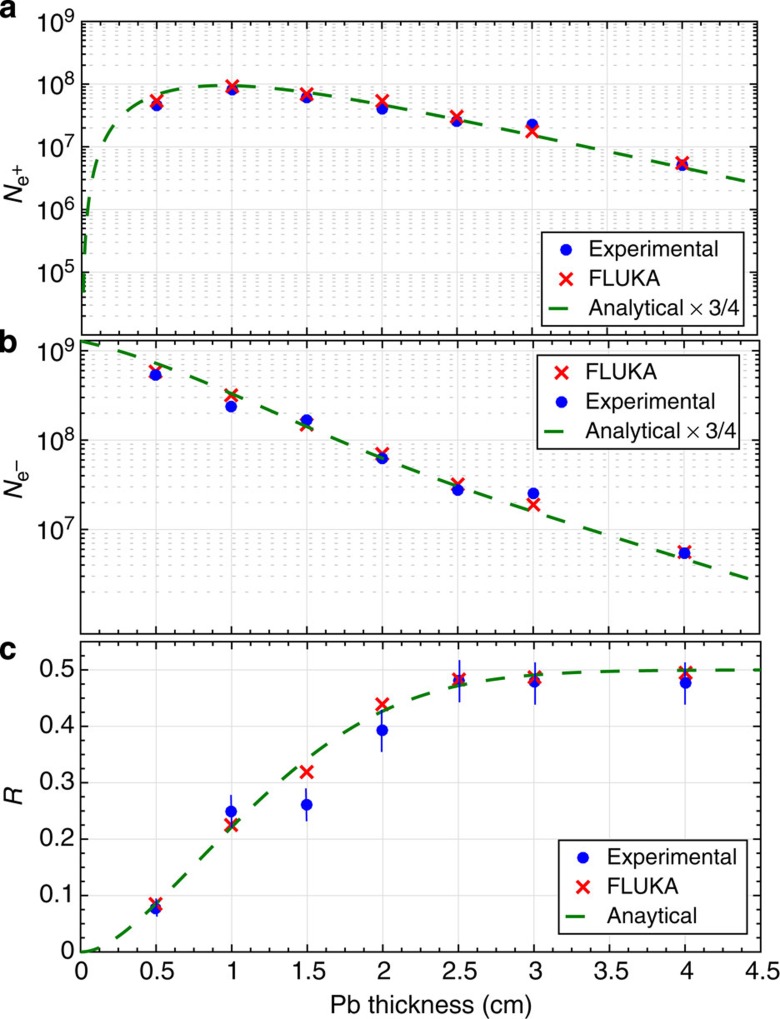
Percentage of positrons in the leptonic beam. (**a**) Measured (blue circles) and simulated (red crosses) number of positrons 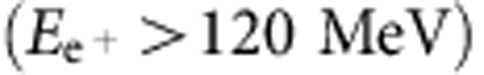 as a function of the Pb thickness (
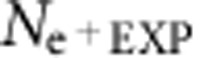
, see main text). The green dashed line represents the analytical prediction (discussed in the text). (**b**) Measured (blue circles) and simulated (red crosses) number of electrons 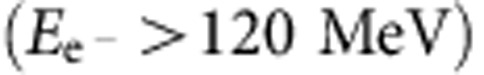 as a function of the Pb thickness (
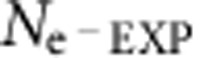
, see main text). The green dashed line represents the analytical prediction (discussed in the text). For these two frames, error bars lie within the size of the circles. (**c**) Percentage of positrons in the leptonic jet: measured (full blue circles), simulated (red crosses) and analytical prediction (green dashed lines). For all panels, the error bars mainly arise from shot-to-shot fluctuations. FLUKA simulations indicate that the overall number of relativistic electrons and positrons (*E*_e_≥1 MeV) behave in a similar manner. The percentage of positrons in the beam reaches ∼50% for *d*>2.5 cm≈5*L*_rad_.

A scan in target thickness was performed in multiples of its radiation length and the obtained positron spectra, each resulting from an average over five consecutive shots, are depicted in Fig. 2 (see Fig. 1c for the raw signal recorded on the LANEX screen for *d*=0.5 cm). All spectra are in good agreement with the ones resulting from matching simulations using the Monte Carlo scattering code FLUKA, which accounts for electromagnetic cascades during the passage of an electron beam through a solid target^26^ (see Methods section). A maximum positron energy of *E*_MAX_=600 MeV is obtained for *d*≈*L*_rad_ (that is, 5 mm; Fig. 2a), whereas a maximum positron yield is obtained for *d*≈2*L*_rad_. For thicker targets, the maximum energy gradually decreases as it should be expected due to increased probability of energy loss during the propagation of the generated positrons through the rest of the solid target. For a similar reason, a thicker solid target allows a lower number of electrons and positrons to escape it. This is quantitatively shown in Fig. 3, which depicts the measured number of electrons and positrons (energy exceeding 120 MeV; see Methods section) at the exit of a solid target, as a function of its thickness.

In order to quantitatively explain the observed trends, we have employed a simple analytical model for a quantum electrodynamic cascade that only includes the emission of photons by electrons and positrons via bremsstrahlung^27^ and the creation of an electron–positron pair by a photon^28^, both processes occurring in the field of a heavy atom. We thus neglect additional energy losses as resulting, for instance, from Compton scattering with the electrons of the atoms and from the ionization of the atoms themselves (see Methods section). This model is able to qualitatively reproduce the experimental trends (dashed green curves in Fig. 3), provided that a constant re-scaling factor of about 0.75 is adopted for the absolute yield of both the electrons and positrons. This overestimate is easily understood, as the semi-analytical model does not take into account a number of energy loss mechanisms, such as Compton scattering and the ionization of atoms^29^. Once this re-scaling factor is applied, the analytical model reproduces the experimental data within a few per cent, clearly indicating that the only processes of bremsstrahlung and electron/positron pair production in the nuclear field are the dominant mechanisms leading to the generation of the detected electron/positron beam.

**Figure 4:**
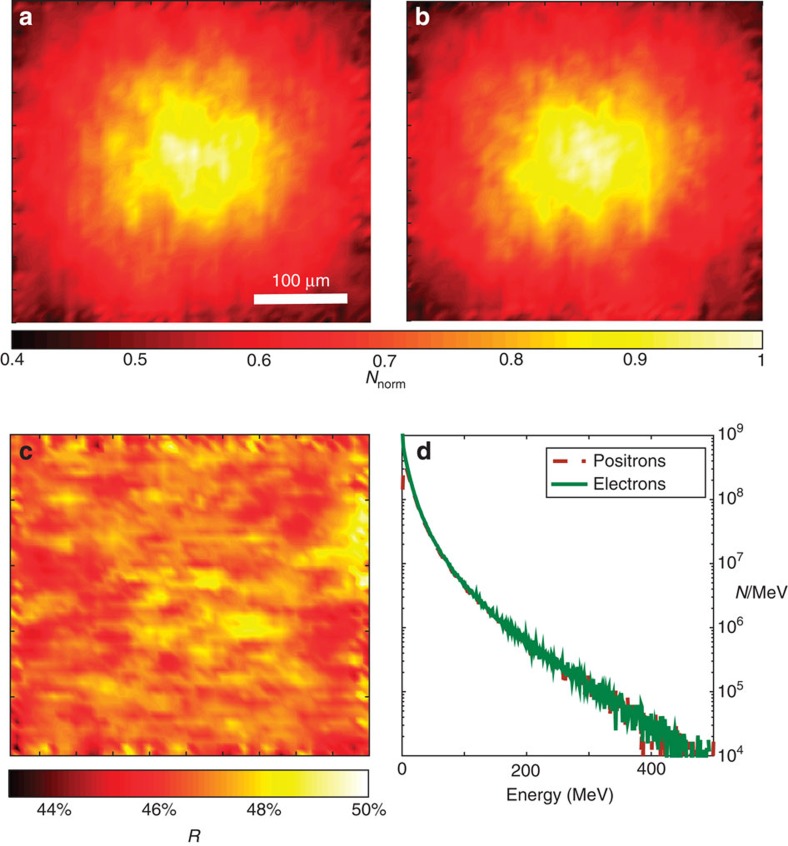
Charge neutrality is spatially uniform across the leptonic beam. Normalized spatial distribution of the electrons (**a**) and positrons (**b**) at the rear surface of a 2.5 cm Pb foil, as simulated using FLUKA (details of the simulation in the Methods section). (**c**) Resulting spatially resolved percentage of positrons in the leptonic beam. The positron percentage oscillates between 45 and 49% across the whole leptonic beam. (**d**) Simulated spectrum of the electrons (solid green line) and positrons (dashed brown line) at the rear surface of the 2.5 cm Pb foil. Charge imbalance is found only in the low-energy part of the spectrum (energy of the order of 5 MeV).

**Figure 5:**
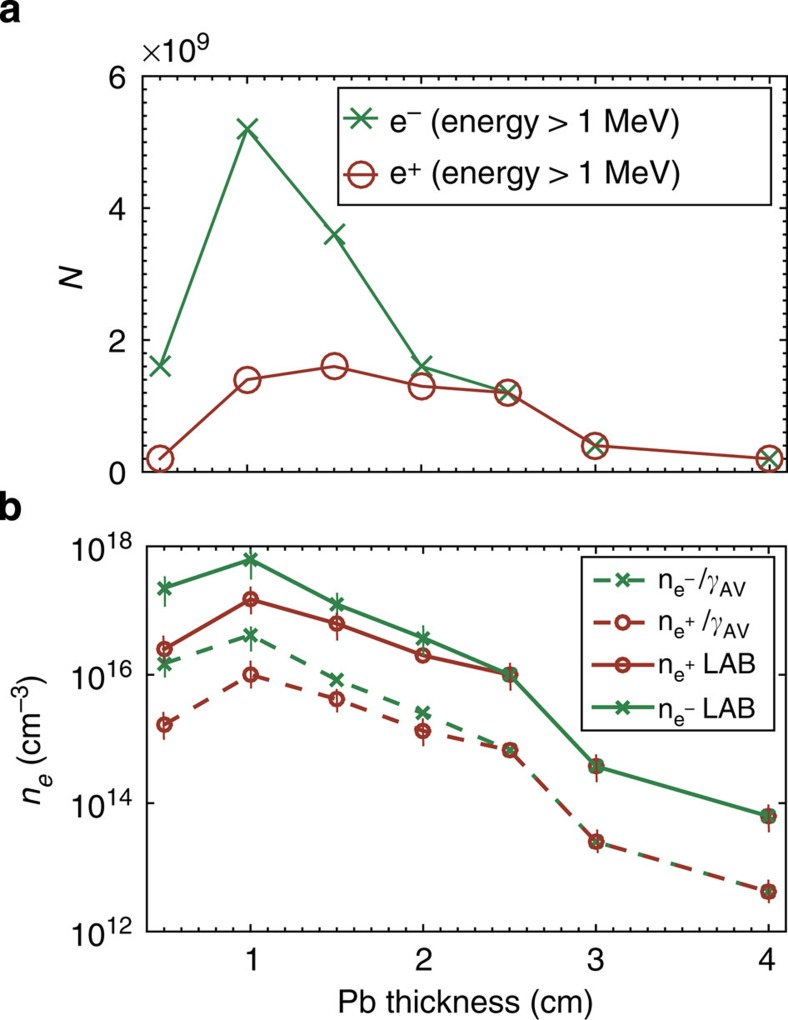
Density of the leptonic beam (**a**) Number of relativistic electrons (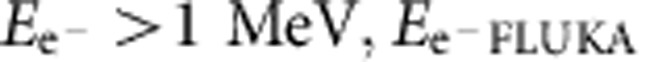, green crosses) and positrons (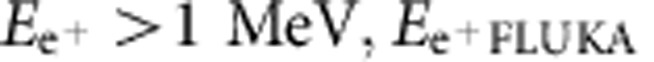, brown circles) in the beam as a function of the thickness *d* of the solid target. (**b**) Density of relativistic electrons (green crosses) and positrons (brown empty circles) as a function of the thickness *d* of the solid target. The solid lines represent the density in the laboratory reference frame, whereas dashed lines represent the beam proper density. A neutral electron–positron plasma is obtained for *d*≥2.5 cm. Lines are drawn only as a guide for the eye.

Let us now turn our attention to the total positron fraction in the leptonic beam 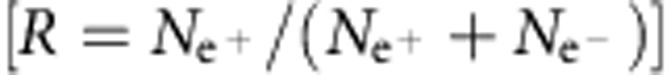 as a function of the target thickness (plotted in Fig. 3c). For *d*≈*L*_rad_, the positrons account for ∼8–10% of the overall beam owing to the fact that most of the primary electrons are able to escape the target (consistently with the results reported in ref. 21). However, as we increase the target thickness, this ratio increases up to a point where the positrons account for almost 50% of the leptonic jet (*d*≥2.5 cm; Figs 3c and 4 for the overall charge imbalance in the leptonic beam and its simulated spatial distribution, respectively). In this case, not only the integrated number of electrons and positrons is similar but also their spectrum (Fig. 2c), further indication that almost all the electrons and positrons escaping the target arise from pair production. A positron percentage in the beam of the order of 50% is preserved also if the target thickness is increased; however, we will focus our attention only on *d*=2.5 cm, since it provides the highest density of the neutral e^−^/e^+^ beam. Simulations confirm that the majority of positrons are generated with energies of the order of a few MeV following a Jüttner–Synge distribution, which is commonly assumed for relativistic thermalized plasmas^30^ (see, as an example, the inset in Fig. 2a). We thus refer to the experimentally measured number of e^−^ and e^+^ 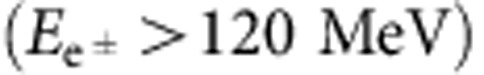 with the subscript *N*_EXP_, whereas we will refer to their simulated number 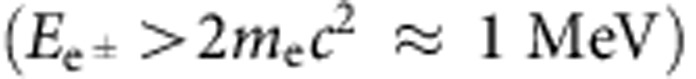 with the subscript *N*_FLUKA_. For *d*=2.5 cm, we thus have 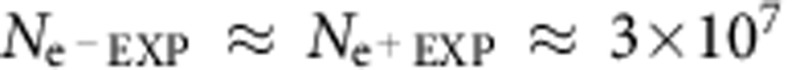 and 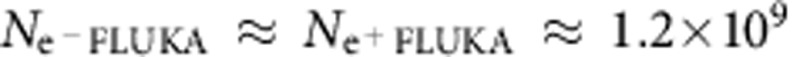 (Fig. 5a). Taking the appropriate moment of the distribution function, the averaged Lorentz factor of the beam is typically of the order of a few tens (*γ*_AV_≈15 for *d*=2.5 cm). FLUKA simulations indicate a divergence of the beam to be energy dependent in a range of 5–20 mrad (ref. 31).

It must be pointed out that the propagation of an ultra-relativistic electron beam through a high-*Z* solid target can only asymptotically give a perfectly neutral e^−^/e^+^ beam. Additional scattering mechanisms with the atomic electrons, such as Compton, Moller and Bhabha scattering, will in fact slightly increase the electrons number, especially at low energies. FLUKA simulations take all these processes into account and indeed predict an average percentage of positrons, for *d*=2.5 cm, of 46%. The discrepancy between electron and positron number is exclusively at low energies (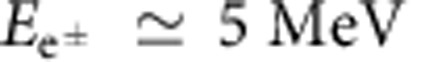; Fig. 4d). Most importantly, the electron and positron populations present very similar spatial distributions (Fig. 4a,b) leading to an almost uniform positron percentage in the e^−^/e^+^ beam (between 45 and 49%; Fig. 4c). As we shall see later, this slight charge imbalance does not affect the plasma dynamics, which can then be effectively considered to be neutral.

A fundamental requisite for the laboratory study of e^−^/e^+^ plasmas is that they must present collective behaviour in their dynamics. Collective (that is, plasma-like) effects are likely to occur in the beam only if its transverse size *D*_B_ is larger than the collision-less skin depth (*l*_skin_≃*c*/*ω*_prop_, with *ω*_prop_ being the relativistic plasma frequency). The beam density is determined by the temporal duration of the beam (that relates to its longitudinal extent) and its transverse size. The primary electron beam exits the gas jet with a typical temporal duration comparable to half the plasma period within the gas^32^: *τ*_pl_≃(13.0±0.3) fs. The semi-analytical model for the quantum cascade inside the Pb indicates an average temporal spreading across different spectral components of the beam of the order of 1–3 fs, resulting in a beam duration of 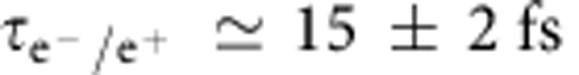. As intuitively expected, the lower energy electrons and positrons will escape the solid target in a wider area if compared with their higher energy counterparts. FLUKA simulations confirm this expectation and indicate, for *d*=2.5 cm, a maximum transverse size of the beam of the order of *D*_B_≃200±30 μm. For these parameters, we thus obtain a particle density in the laboratory reference frame of the order of *n*_e_≃(1.8±0.7) × 10^16^ cm^−3^, implying a beam proper density of *n*_prop_=*n*_e_/*γ*_AV_≃(1.5±0.5) × 10^15^ cm^−3^ (Fig. 5b). The relativistically corrected collision-less skin depth of the beam is thus *l*_skin_≃*c*/*ω*_prop_(160±30) μm. This value is smaller than the beam transverse size, indicating that the generated particle beam is a neutral e^−^/e^+^ plasma. It is interesting to note that the occurrence of collective behaviour (that is, the situation in which *D*_B_/*l*_skin_≥1) does not depend on the beam transverse size *D*_B_ since, based on the considerations presented above, it can be expressed as: 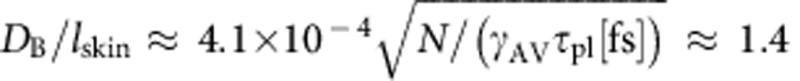 for our experimental parameters (here *N* indicates the overall number of leptons in the beam).

## Discussion

The presented characteristics of the e^−^/e^+^ plasmas generated in our experiment are appealing for the laboratory study of the dynamics of this exotic state of matter. As an example, a particularly active area of research in this direction is the determination of the growth and evolution of kinetic instabilities, which are extensively modelled in order to interpret peculiar astrophysical observations such as the emission of gamma-ray bursts^33^,^34^,^35^,^36^. It is widely accepted that these ultra-bright bursts result from synchrotron radiation generated via relativistic shocks triggered during the propagation of an electron–positron beam through the low-density intergalactic medium^37^. This scenario is now reproducible in a laser-driven experiment in which the photoionized residual low-density gas inside the target chamber^38^ can act as the background electron-ion plasma. In this case, the growth rate for transverse instabilities can be estimated as: 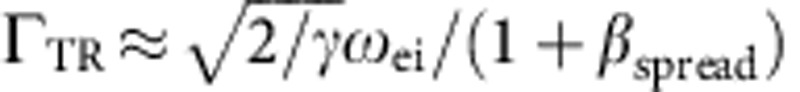 (ref. 13), with *β*_spread_ and *ω*_ei_ being the velocity spread of the e^−^/e^+^ beam and the plasma frequency of the e^−^-ion plasma, respectively. It is worth noticing that in the ultra-relativistic case, the weak dependence of the growth rate on the beam velocity spread significantly relaxes constraints on the spectral shape of the electron–positron beam. We can assume *ω*_ei_≈1.5 × 10^12^ Hz (*n*_ei_≈6 × 10^14^ cm^−3^ as resulting from full photoionization of the background gas) and *β*_spread_≈0.1 (*β*=0.87 and *β*≈1 for a 1 MeV and a 500 MeV particle, respectively). We thus have Γ_TR_=5 × 10^11^ Hz for *γ*_AV_=15 implying a typical time for the instability to grow of the order of 2 ps. Numerical simulations indicate, in the initial instants of the instability, that up to 10% of the average particle energy in the beam can be transformed into electromagnetic fields in the plasma implying fields with an amplitude of the order of the megagauss; once saturation is reached, this value drops to ∼1% (ref. 13). It is worth noticing that this is similar to what expected for gamma-ray bursts (0.1–1%; ref. 39). This timescale and field amplitude are within reach of plasma radiography techniques such as proton imaging^40^, a highly encouraging factor for the application of these plasmas for laboratory astrophysics.

**Figure 6:**
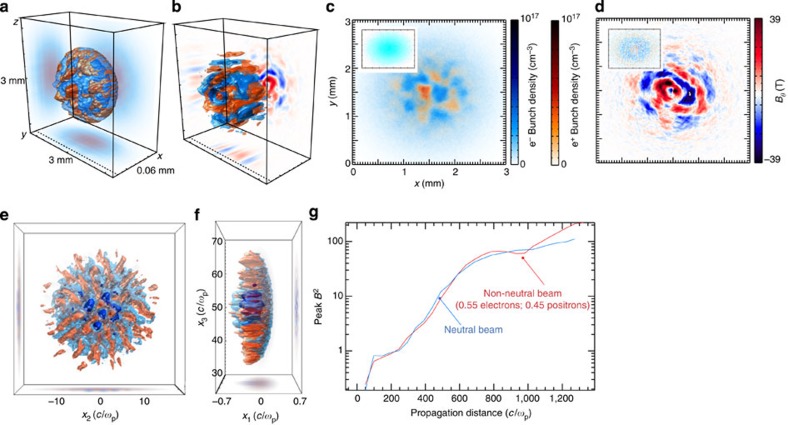
PIC simulations of the leptonic beam dynamics in a background electron-ion plasma Simulation results of the propagation of an e^−^/e^+^ plasma through an e^−^-ion plasma. The first row depicts the results for a perfectly neutral beam (50% electrons and 50% positrons). (**a**) Electron (blue) and positron (red) density isosurfaces showing growth of the Weibel instability at the back of the bunch. (**b**) Magnetic field (*B*_θ_) filaments due to the Weibel instability, where the *B*_θ_ lies on the plane transverse to bunch propagation direction. (**c**) Fireball bunch density slice taken at the position of the dashed line in **a**. (**d**) Corresponding magnetic *B*_θ_ filaments taken at the same location. The insets in **c**,**d** show the electron density and magnetic field corresponding to the propagation of a purely electronic beam showing no onset of filamentation. Frames (**e**,**f**) depict the results for an analogous simulation, with the only difference that now the positrons account only for 45% of the beam. The frames show slices of the electron (blue) and positron (red) spatial distribution, similar to frame **a**. (**g**) Comparison between the magnetic field growth for the case of a purely neutral beam (blue) and a slightly asymmetric beam (45% of positrons, red). For what concerns the plasma dynamics, the two cases are virtually undistinguishable.

In order to check the validity of our estimates, we have carried out three-dimensional (3D) particle-in-cell (PIC) simulations using the PIC code OSIRIS^41^,^42^ (see Methods section). Simulation results are illustrated in Fig. 6. During its propagation through a denser e^−^-ion plasma, the e^−^/e^+^ is subject to the Weibel/current filamentation instability leading to the formation of electron and positron filaments with thicknesses of the order of the beam skin depth. The electron and positron filaments spatially separate from each other leading to net localized currents and the generation of the corresponding azimuthal magnetic field structures with maximum amplitudes of the order of 40 T in the middle of the bunch. At early times, the simulations show that the transverse scale length of the filaments is even shorter than the initial beam skin depth. To further understand the impact of charge neutrality on the instability onset, additional 3D simulations were performed using a purely electronic bunch of same characteristics. In this case, the electron bunch generates plasma wakefields, and neither filamentation of the beam (insets in Fig. 6c) nor the generation of strong magnetic fields (inset Fig. 6d) are observed. These results corroborate the expectation that current filamentation instability growth can be controlled by changing the beam overall total charge and it is maximized for a purely neutral e^−^/e^+^ plasma.

Finally, we performed an additional 3D PIC simulation devoted at studying whether a slight charge imbalance in the e^−^/e^+^ plasma could result in a change in the plasma dynamics if compared with the idealized perfectly neutral plasma scenario. We have thus maintained exactly the same conditions as the other simulation, with the only difference that now the positron account for 45% of the plasma population, in order to match our experimental findings more closely. The obtained spatial distribution of the e^−^/e^+^ plasma after propagation through the background electron/ion plasma is shown in Fig. 6e,f, indicating essentially no difference if compared with the purely neutral case. This statement is corroborated by the growth of magnetic fields due to Weibel instability. This is plotted in Fig. 6g that shows virtually the same magnetic field growth for the purely neutral case (blue line) and for the slight charge imbalance (red). For the point of view of studying electron–positron plasma dynamics in the laboratory, the e^−^/e^+^ plasma generated in our experiment is virtually indistinguishable from the idealized purely neutral beam.

On the other hand, the beam might also be susceptible to longitudinal instabilities^34^,^43^, which would induce a broadening of the e^−^/e^+^ spectrum and generation of strong fields in the background plasma. For *d*=4 cm (neutral beam), the measured electron and positron spectra are indeed flatter than the ones predicted by FLUKA, which does not include collective behaviour of the beam particles during propagation through the background e^−^-ion plasma (Fig. 2c). For *d*=0.5 cm (highly charged beam), simulations and experiments agree much more closely. The spectral flattening may also be produced by kinetic self-focusing of the beam^44^,^45^.

In conclusion, we have reported on the first creation of a neutral electron–positron plasma in the laboratory. Its overall charge neutrality and plasma-like behaviour are an absolute novelty in the field of experimental physics and, in conjunction with the small divergence and high energy of these plasmas, finally allow for the laboratory study of this unique state of matter.

## Methods

### The electron–positron spectrometer

The magnetic spectrometer comprised a pin-hole entrance with a diameter of ∼15 mm through 5 cm of plastic followed by 5 cm of lead. This plastic–lead wall was indeed necessary in order to shield the particle detectors from noise generated during the electron beam impact onto the solid target. After this, a dipole permanent magnet (*B*=0.8 T, length of 10 cm) was inserted to spectrally resolve the electrons and the positrons, which were recorded by two LANEX screens^46^. This arrangement allowed us to resolve particle energies from 120 MeV to 1.2 GeV. The LANEX screens were cross-calibrated using absolutely calibrated Imaging Plates^47^. The small difference in stopping power (of ∼2%; ref. 48) between electrons and positrons was taken into account in calibrating the LANEX screens. Every electron or positron spectrum shown in the manuscript results from an average over five consecutive shots. The energy resolution of the spectrometer can be approximated in the ultra-relativistic limit, as:


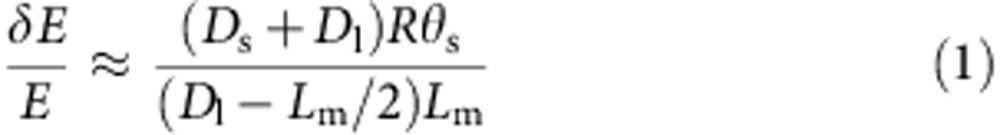


Where *D*_s_ is the distance from the source to the magnet entrance, *D*_l_ is the distance from the entrance of the magnet to the detector (1 m), *R*_L_≈*E*/(*ecB*) is the radius of curvature of the particle with energy *E* and charge *e* in the magnetic field *B*, *θ*_s_=15 mrad is the angular acceptance of the detector, and *L*_m_ (10 cm) is the length of the magnet. For the energies of interest in our experiment (120≤E[MeV]≤300), the energy resolution is between 10 and 20%.

### FLUKA simulations

FLUKA is a nuclear physics Monte Carlo scattering code that accounts for electromagnetic cascades during the passage of an electron beam through a solid target^26^. The numerical model for the quantum electromagnetic cascade is routinely checked and constantly improved to take into account any refinement in cross-section measurements in conventional accelerators. As an input for the simulation, we assume an electron beam with the spectral shape depicted in Fig. 1b (brown solid line), 2 mrad full-width half-maximum divergence and 10 μm radius source size. The electron beam then interacts with a lead target of different thicknesses and 1 cm transverse size, placed 1 cm downstream of the electron beam source. Iterations (10^6^) were used in order to achieve a good statistical representation in the Monte Carlo method. Every numerical result reported originates from an average over five identical runs in order to minimize any stochastic error arising from the random seed generator of the code. The results of the simulations, obtained in units of particles per initial electron, were then rescaled with the measured number of primary electrons, giving a good quantitative agreement with the experimental data.

### Semi-analytical model for the quantum cascade

We assume a quantum electrodynamics cascade shower involving only electrons, positrons and photons at energies much larger than the electron rest energy *m* (units with *h*=*c*=1 are assumed hereafter). We thus neglect additional electron and positron energy losses as resulting, for instance, from Compton scattering with the electrons of the atoms and from the ionization of the atoms themselves. The only processes to be included in the kinetic equations are thus the emission of photons by electrons and positrons via bremsstrahlung and the creation of an electron–positron pair by a photon, both processes occurring in the field of a heavy atom. By setting the target thickness *d* in units of the radiation length *L*_rad_, that is, ℓ=*d*/*L*_rad_, the electron/positron distribution functions *f*_±_(*E*,ℓ) and the photon distribution function *f*_γ_(*E*,ℓ) satisfy the kinetic equations^29^:


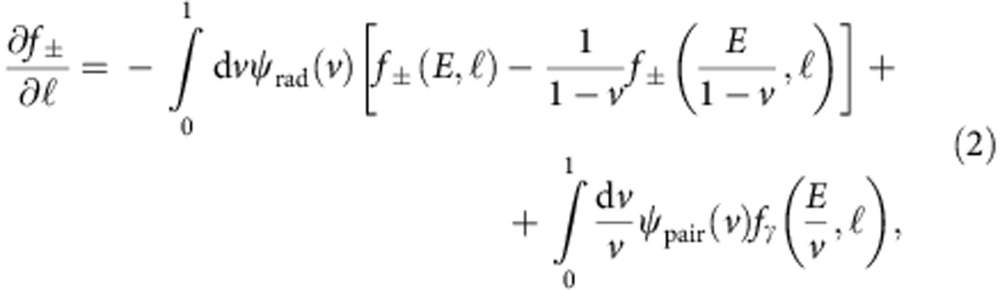



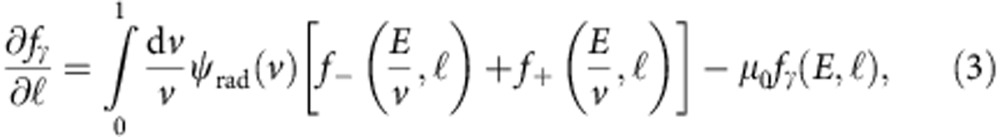


where the functions


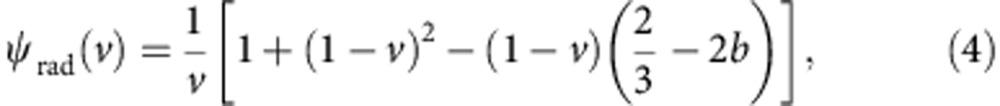



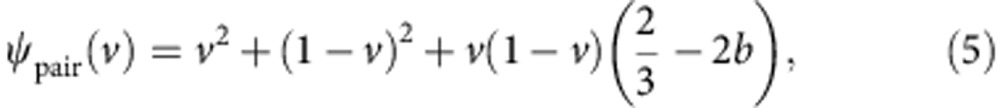


with *μ*_0_=7/9−*b*/3 and *b*=1/18 log(183/*Z*^1/3^), are related to the cross-section of bremsstrahlung and pair photo-production in the field of a heavy atom with atomic number *Z* (see ref. 29 for details). By numerically solving these equations, we are able to reproduce the experimental trends well (dashed green curves in Fig. 3 of the manuscript), provided that a constant re-scaling factor of 0.75 is adopted for the absolute yield of both the electrons and positrons. The overestimation of the experimental results by this simplified model is easily understood, as the latter does not take into account a number of braking mechanisms, such as Compton scattering for photons and the ionization of atoms for electrons and positrons^29^. On the one hand, braking mechanisms such as ionization affect essentially relativistic electrons and positrons in the same way^49^. On the other hand, however, our analytical model cannot predict charge asymmetries brought in by injection of atomic electrons in the cascade following these scattering processes. This is the reason why, for a target thickness of 2.5 cm, our semi-analytical model predicts a 50% percentage of positrons in the leptonic beam, whereas our FLUKA simulations indicate a positron percentage of the order of 46%.

Starting from a simple model, where each electron/positron (photon) after a radiation length emits a photon (transforms into an electron–positron pair) with half of the energy of the initial electron (with the electron and positron sharing half of the energy of the initial photon), it can also be shown that the maximum yield of positrons with an energy exceeding *E* can be estimated to occur for a target thickness *d*_opt_∼*L*_rad_ log(〈*E*_e_〉/*E*)/log(2) (ref. 29), where 〈*E*_e_〉 is the average energy of the initial electron distribution (Fig. 1b). In our case, it results 〈*E*_e_〉≈456 MeV and *d*_opt_∼1.1 cm (≃1.96 *L*_rad_) in good agreement with the experimental results.

### The PIC simulations

The simulations were performed with the fully relativistic, massively parallel, PIC code OSIRIS^41^,^42^. OSIRIS has been extensively used to explore relativistic beam plasma interaction scenarios, and has been widely applied to model the Weibel instability in various configurations (see, for instance, refs 13, 14, 41, 42, 50). In OSIRIS, the electric and magnetic fields are defined in a grid. The trajectory of each simulation particle is determined through the relativistic equations of motion by interpolating the grid fields to the position of the particle. Current density is deposited onto the grid, and used to advance the electric and magnetic fields through Maxwell's equations discretized using a finite-difference scheme. In this section, we give the numerical parameters for the simulations. Simulations used a moving window with dimensions 1.5 × 100 × 100 (*c*/*ω*_p_)^3^ divided into 75 × 1,000 × 1,000 cells with 2 × 1 × 1 particles per cell for plasma electrons and for beam particles. Here *ω*_p_ is the plasma frequency of the background electron–proton plasma, which has a density of *n*_ei_=10^16^ cm^−3^. A charge–neutral beam constituted by electrons and positrons was initialized at the entrance of the plasma. The density profile for electrons and positrons is given by 
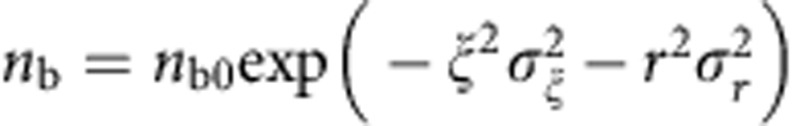
 where *n*_b0_=10 *n*_ei_=10^17^ cm^−3^, *σ*_*ξ*_=0.22 *c*/*ω*_p_=11.7 μm and *σ*_*r*_=10 *c*/*ω*_p_=530 μm are the bunch peak density, length and transverse waist, respectively. The particles' Lorentz factor is initialized to be 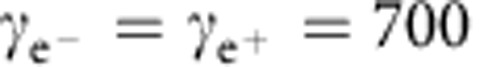.

## Additional information

**How to cite this article:** Sarri, G. *et al*. Generation of neutral and high-density electron–positron pair plasmas in the laboratory. *Nat. Commun*. 6:6747 doi: 10.1038/ncomms7747 (2015).
